# The Effects of Gas Saturation of Electrolytes on the Performance and Durability of Lithium‐Ion Batteries

**DOI:** 10.1002/cssc.202100845

**Published:** 2021-06-16

**Authors:** Lars Bläubaum, Philipp Röse, Leon Schmidt, Ulrike Krewer

**Affiliations:** ^1^ Institute for Applied Materials – Electrochemical Technologies Karlsruhe Institute of Technology Adenauerring 20b 76131 Karlsruhe Germany; ^2^ Institute of Energy and Process Systems Engineering Technische Universität Braunschweig Langer Kamp 19b 38106 Braunschweig Germany

**Keywords:** batteries, carbon dioxide, charge transfer process, electrolytes, solvation effects

## Abstract

Traces of species in batteries are known to impact battery performance. The effects of gas species, although often reported in the electrolyte and evolving during operation, have not been systematically studied to date and are therefore barely understood. This study reveals and compares the effects of different gases on the charge‐discharge characteristics, cycling stability and impedances of lithium‐ion batteries. All investigated gases have been previously reported in lithium‐ion batteries and are thus worth investigating: Ar, CO_2_, CO, C_2_H_4_, C_2_H_2_, H_2_, CH_4_ and O_2_. Gas‐electrolyte composition has a significant influence on formation, coulombic and energy efficiencies, C‐rate capability, and aging. Particularly, CO_2_ and O_2_ showed a higher C‐rate capability and a decrease in irreversible capacity loss during the first cycle compared to Ar. Similar discharge capacities and aging behaviors are observed for CO, C_2_H_4_ and CH_4_. Acetylene showed a large decrease in performance and cycle stability. Furthermore, electrochemical impedance spectroscopy revealed that the gases mainly contribute to changes in charge transfer processes, whereas the effects on resistance and solid electrolyte interphase performance were minor. Compared to all other gas–electrolyte mixtures, the use of CO_2_ saturated electrolyte showed a remarkable increase in all performance parameters including lifetime.

## Graphical abstract

**Gas saturation matters**: Saturation of lithium‐ion battery electrolytes with various gases is systematically investigated. Significant differences in cell performance, C‐rate capability and charge transfer processes are identified between different gases. Depending on the gas and operating conditions, a trade‐off between performance increase or loss and aging behavior is observed. The best result is obtained for CO_2_‐saturated electrolytes.

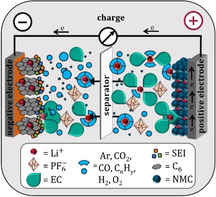

## Introduction

**Figure 1:**
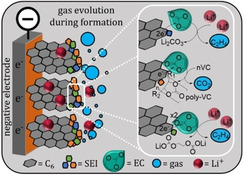
Evolution of gases by electrolyte decomposition on the negative electrode surface during the first cycle. The dissolved gases remain in the electrolyte after the formation and may thus impact performance; the white box displays selected gas evolving reactions.

Electrolytes are essential components for all electrochemical energy storage technologies. Since the electrolyte is in contact with almost all components and interacts with them during electrochemical operation, the tailoring of electrolyte composition is crucial to achieving high performance. This holds especially true for lithium‐ion batteries as they are very sensitive to changes in formulation.^[1]^ Therein, organic electrolytes take a key role in terms of performance parameters such as the working temperature range, power density, high‐rate capability, cycle life and the safety of the whole system.[^2^, ^3^, ^4^, ^5^] Intensive efforts have been made to identify the best suited electrolyte compositions. Most electrolytes contain mixtures of aprotic organic solvents such as ethylene carbonate (EC), ethyl methyl carbonate (EMC), dimethyl carbonate (DMC) and diethyl carbonate (DEC), in combination with an inorganic conductive salt LiPF_6_. Beside these components, additives such as vinylene carbonate (VC) or cyclohexyl benzene (CHB) are added to provide enhanced performance or to increase safety.[^6^, ^7^] A detailed summary of electrolyte compositions and their impacts on the performances and behaviors of the individual components in lithium‐ion batteries has already been addressed.[^7^, ^8^, ^9^, ^10^] However, the workings of electrolytes saturated with gases that typically form during formation and operation, or that may even come from the manufacturing process, have not yet been systematically investigated. This is astonishing, as it is well known that lithium‐ion battery components undergo significant changes in morphology and composition during their formation due to chemical reactions or decomposition of the electrolyte where several gases are produced (Figure 1).

The (electro‐)chemical reactions at the electrode/electrolyte interface of the negative electrode (and thus the produced gases) are induced by the low operating potential of the negative electrode which is close to or beyond the stability window of the electrolyte.^[11]^ Here, a range of different gases is formed [see Eqs. (1)–(6)].[^12^, ^13^, ^14^, ^15^, ^16^, ^17^, ^18^] These reactions are desired to a certain degree, since they result in the formation of the solid electrolyte interphase (SEI) which is a protective layer that covers the negative electrode surface and ensures reversible intercalation of lithium ions into the graphite electrode while preventing further electrolyte degradation.[^19^, ^20^, ^21^, ^22^, ^23^, ^24^, ^25^] The formation of important SEI components, such as lithium alkyl carbonates [e. g., LEDC; Eq. (1)], lithium alkoxides [Eq. (2)], and polymers [Eq. (3)], also results in ethylene, carbon monoxide, and carbon dioxide evolution. These preliminary SEI components may react further to form more stable SEI species, such as Li_2_CO_3_, Li_2_O, or LiF, and additional gases [Eq. (4)].[^19^, ^20^, ^21^, ^22^, ^23^] Furthermore, gases may evolve during aging, depending on the composition of the cell. For example, when using cobalt‐containing electrodes, O_2_ may evolve [Eq. (5)], and when using a binder such as PVdF, H_2_ may evolve [Eq. 6].

(1) $$2\;C_{3}H_{4}O_{3}\;\left(\mathrm{EC}\right)\;\overset{+2\;Li^{+},\;2e^{-}}{\underset{}{\to }}\;(CH_{2}\mathrm{OC}O_{2}\mathrm{Li}{)}_{2}\;+\;C_{2}H_{4}↑$$

(2) $$C_{3}H_{6}O_{3}\;\left(\mathrm{DMC}\right)\;\overset{+2\;Li^{+},\;2e^{-}}{\underset{}{\to }}\;2\;\mathrm{LiOC}H_{3}\;+\;\mathrm{CO}↑$$

(3) $$C_{3}H_{2}O_{3}\;\left(\mathrm{VC}\right)\;\overset{+e^{-},\;n\;\mathrm{VC}}{\underset{}{\to }}\;[\mathrm{VC}{]}_{n+1}\;+\;CO_{2}↑$$

(4) $$(CH_{2}\mathrm{OC}O_{2}\mathrm{Li}{)}_{2}\;(\mathrm{LEDC})\to Li_{2}CO_{3}\;+C_{2}H_{4}↑+\;CO_{2}↑+\;\frac{1}{2}\;O_{2}↑$$

(5) $$Li_{0.5}\mathrm{Co}O_{2}\to \frac{1}{2}\;\mathrm{LiCo}O_{2}\;+\;\frac{1}{6}\;Co_{3}O_{4}\;+\;\frac{1}{6}\;O_{2}↑$$

(6) $$[CH_{2}-CF_{2}{]}_{n}\;(\mathrm{PVdF})\;\overset{+n\;\mathrm{Li}}{\underset{}{\to }}\;n\;\;\mathrm{LiF}+[\mathrm{CH}=\mathrm{CF}{]}_{n}+\frac{n}{2}\;H_{2}↑$$

Despite so many evolving gases, most have not yet been considered to have any influence on the performance of lithium‐ion batteries. This is contrary to research on lithium metal batteries, where vast challenges lie in controlling the metal/electrolyte interface reactions and properties. Here, lithium reportedly reacts spontaneously with most atmospheric gases and (depending on their compositions) different reaction pathways may occur. In particular, the behaviors of CO_2_, CO, O_2_, N_2_ and N_2_O in the electro‐deposition of lithium on nickel and lithium foil were intensively investigated. It has been reported that the addition of those gases leads to the formation of a homogenous layer of inorganic lithium salts.[^26^, ^27^, ^28^, ^29^, ^30^] Whereas almost all added gases had effects on cell performance, the best results were achieved from CO_2_ addition.[^31^, ^32^] The reaction of CO_2_ with lithium resulted in the formation of Li_2_CO_3_ which is enriched in the inner layer of the surface film on the lithium negative electrode. In the absence of CO_2_, only traces of Li_2_CO_3_ were found on lithium electrodes. The presence of Li_2_CO_3_ produced a smoother surface and lower overpotential. Likewise, a lower interfacial resistance was observed, and lithium dendrite formation was further suppressed. This resulted in an enhanced cycling efficiency, C‐rate capability and long‐term stability of the negative electrode materials.[^27^, ^32^, ^33^, ^34^, ^35^, ^36^, ^37^] Moreover, Strehle et al. discovered that gases, such as CO_2_, are probably responsible for suppressing the transesterification of EMC.^[38]^ Similar results were achieved by adding Li_2_CO_3_ to the electrolyte solution.[^39^, ^40^] Shiraishi et al. reported that treating lithium foil with mineral acids gave a thin bilayer surface structure with Li_2_O at the inner surface and further lithium salts at the outer surface/electrolyte interface.^[41]^ In conclusion, gases play a crucial role in lithium metal electrodes, and might also do so in lithium‐ion batteries. This is highly likely, as many of the decomposition reactions either involve lithium metal directly (corrosive) or utilize lithium ions from the solution (non‐corrosive).^[26]^ This paper is the first systematic study to shed light on the effects of typical formation gases on the performance and lifetime of lithium‐ion cells.

There are different methods of analyzing the gases and their impacts on lithium‐ion batteries. Researchers can simulate the direct impact of gases on surface film growth in multiscale film growth models that contain complex reaction networks,^[42]^ however parameterization may be challenging. The effects may also be studied experimentally using discharge curves and especially dynamic electrochemical measurements to separate impacts on reaction, transport and SEI processes, which cause different performance losses. Electrochemical impedance spectroscopy (EIS) is often used to quantify SEI properties during aging.^[43]^ Moreover, even more sensitive to reactions and degradation at the surface is the nonlinear version of impedance spectroscopy, nonlinear frequency response analysis.^[44]^ Among other advantages, it enables one to distinguish between plating and cycling aging.[^45^, ^46^] However, even today this method is not easy to interpret. Thus, for an initial study, we used EIS and discharge curves at different currents to study the impact of gases on performance. This enabled us to attribute changes in performance and lifetime to changes in the cell transport and kinetic losses. To clearly understand the impact of the individual gases, they will be externally introduced to the cell by saturating the electrolyte before cell assembly. Charge–discharge characteristics, C‐rate capability, efficiencies and EIS are shown to give a detailed understanding and quantification of gas impacts. The study thus paves the road for future tailoring of electrolyte pretreatment of lithium‐ion batteries to increase performance and lifetime.

## Results and Discussion

In the following, we first reveal the gas impacts on the discharge capacity during cycling to give a general overview on the impact of the individual gases on battery performance and lifetime. Subsequently, we analyze the discharge curve itself, including reference half‐cell measurements, the C‐rate performance, and the impedance spectra.

### Discharge capacity during cycling

**Figure 2:**
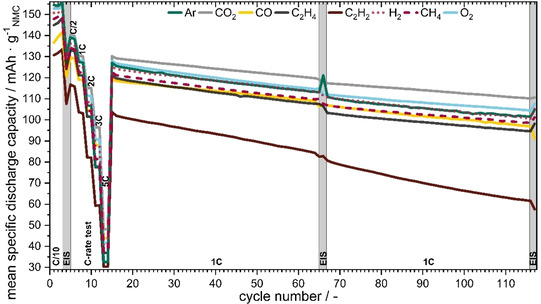
Mean specific discharge capacity vs. cycle number with C‐rate variations and effect of EIS measurement at open circuit potential for the cells with differently gas‐saturated electrolyte. The mean standard deviations, depending on the 1C cycle numbers, amounted to roughly ±2 %, except for C_2_H_2_ with ±11 %.

In a first step, the discharge capacities for all cycles are analyzed to clarify whether the gas‐saturated electrolytes have a significant influence on the cell performance and stability. Figure 2 shows the observed evolution of discharge capacities during formation, C‐rate test, and the two sets of cycling (both sets run for 50 cycles at 1C, before and after EIS measurement). For large differences in the curves, it is clearly visible that gases have a strong impact on performance and that this impact strongly depends on the individual gases. Significant differences between the gases in terms of discharge capacity can already be observed during the formation. The gases CO, C_2_H_4_ and CH_4_ lead to almost similar 1C discharge capacities which were around 3–7 % below the capacities obtained for Ar. Since Ar is typically assumed to be the most neutral and non‐reactive of the gases, it is thus a good reference to see if other gases have a positive or negative impact. In a first conclusion therefore, it seems that CO, C_2_H_4_ and CH_4_ lead to minor declines in 1C performance and lifetime. In contrast, H_2_ exhibits similar behavior to Ar. Finally, CO_2_ and O_2_ saturated electrolytes showed the best performance (i. e., they led to higher capacities). In the 116th cycle, electrolytes saturated with CO_2_ and O_2_ lead to 9 % and 3 % more discharge capacity, respectively, when compared to the Ar reference. Consequently, both performance and lifetime are notably enhanced due to saturation of electrolyte with CO_2_ and O_2_. This is in direct contrast to the cells with a C_2_H_2_ saturated electrolyte, which showed 39 % lower capacity in the 116th cycle than for Ar, and a much faster decrease of cycle stabilities. Thus, C_2_H_2_ should be avoided to obtain well performing cells.

An explanation for such significant differences in discharge capacity and lifetime might be that the different gases promote different SEI formation reactions [Eqs. (1)–(4)], which thus result in a different composition and SEI performance. In addition to the aforementioned frequently reported SEI reactions, CO_2_‐ and O_2_‐saturated electrolytes might cause the formation of lithium peroxide and lithium super oxide, as shown in Equations (7) and (8).^[47]^ Those oxide species are known to have a positive effect on SEI performance.[^27^, ^32^, ^33^, ^34^, ^35^, ^36^, ^37^] Moreover, the low performance of C_2_H_2_ could be due to a polymerization [Eq. (9)], which might lead to an insufficiently conductive SEI.^[48]^ At the same time, Kumai et al.^[13]^ and Kong et al.^[14]^ reported that DMC and hydrogen, as well as ethylene, can react with the positive electrode [Eqs. (10) and (11)]. However, the reactions occurred preferentially upon overcharge. Therefore, we attribute the electrochemical effects we observed mainly to the electrolyte and negative electrode.[^12^, ^49^]

(7) $$CO_{2}\;\overset{+4\;Li^{+},\;4e^{-}}{\underset{}{\to }}2\;Li_{2}O\;+\;C\;$$

(8) $$O_{2}\;\overset{+2\;Li^{+},\;2e^{-}}{\underset{}{\to }}Li_{2}O_{2}$$

(9) $$n\;C{}_{2}H{}_{2}\to \left[C{}_{2}H{}_{2}\right]{}_{n}$$

(10) $$C_{3}H_{6}O_{3}\;\left(\mathrm{DMC}\right)\;+\;H_{2}\;\overset{+2\;Li^{+},\;2e^{-}}{\underset{}{\to }}\;Li_{2}CO_{3}\;+\;2\;CH_{4}↑$$

(11) $$C_{2}H_{4}\to C_{2}H_{2}\;+\;2\;H^{+}\;+\;2\;e^{-}$$

To better understand the possible effects and to narrow down their causes and location, we analyzed the negative electrodes′ discharge and charge behavior including the potential progression and the impedance spectra.

### Cell formation behavior

**Figure 3:**
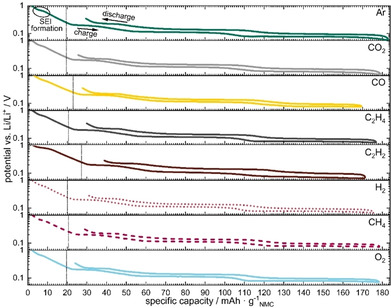
Negative electrode potential vs. charge–discharge capacity, first C/10 formation step for cell electrolytes saturated with different gases; the vertical dotted line indicates the start of lithium intercalation at 0.25 V and the circle in the first graph indicates the phase of the main SEI formation.

To clarify whether the above observed gas impact is caused by a difference in SEI formation, the focus is now turned onto the impact of gas saturation on the first charge and discharge during formation. The respective potential curves provide valuable information about the onset of the initial reactions taking place, as well as side reactions such as lithium loss. The potentials of the negative electrodes during the first formation step of the eight different dissolved gases are shown in Figure 3 (for the potentials of the positive electrode, see Figure S1 in the Supporting Information). It can be seen that gas‐saturated electrolytes lead to significantly different, gas‐specific potential behaviors. Before intercalation started at about 0.25 V vs. Li/Li^+^,^[50]^ the cells consumed different amounts of electric charge for SEI formation (as shown by the vertical lines in Figure 3).

Similar consumptions of electric charges (ca. 20 mAh g^−1^) were found for Ar, CO_2_, C_2_H_4_, H_2_, CH_4_ and O_2_, whereas CO saturated electrolyte consumed about 3 mAh g^−1^ more before a first intercalation took place. The largest amount of electric charge, roughly 40 % more than for Ar, was needed for C_2_H_2_, with about 28 mAh g^−1^. The different fractions in electric charges above 0.25 V vs. Li/Li^+^ indicate for CO and C_2_H_2_ that the gases trigger more (or different) reactions which take place during initial SEI formation.

The SEI potential curve was again similar for all gases, excluding C_2_H_2_. SEI formation was assumed to start when a first plateau is visible;^[51]^ it initiated at approximately 0.7 V for all gases except for C_2_H_2_, which started 0.2 V earlier at 0.9 V. C_2_H_2_, with its highly reactive triple bond, has the lowest reaction barrier and thus reacts non‐specifically with all possible reaction partners in the cell, leading to a complex and unfavorable SEI composition. Furthermore, the gases lead to deviations during lithium intercalation. In general, a narrow gap between charge and discharge potential progression results in lower energy losses due to lower overvoltage. To quantitatively analyze these effects, coulombic efficiencies (CE) and energy efficiencies (EE) were calculated.

**Figure 4:**
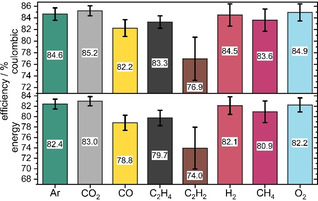
Coulombic and energy efficiencies of first formation cycle (C/10) including standard deviation for cells operated with electrolytes with different gas saturations.

In Figure 4, the coulombic and energy efficiencies of the first charge–discharge cycle are shown for cells operated with different gas‐saturated electrolytes. The gases can be divided into three different classes in terms of coulombic efficiency: 1) Ar, CO_2_, H_2_ and O_2_ with high CE (>84 %); 2) CO, C_2_H_4_ and CH_4_ with slightly lower CE (ca. 83 %); and 3) C_2_H_2_ (with 77 % CE). The differences in CE need to be attributed to different reduction reactions, which change the composition or thicknesses of the surface films leading to different performances.

Likewise, the energy efficiencies (EE) follow a similar trend to the CE of the gases, whereby CO_2_ shows the highest efficiency (83 %). For Ar, H_2_ and O_2_ the EE are similar (ca. 82 %) for the first cycle, due to very similar progression of voltage during the discharge and charge process. In contrast, lower EE were found for CO, C_2_H_4_, CH_4_ (ca. 80 %) and C_2_H_2_ (74 %). Among the electrolytes, CO_2_ showed the highest coulombic and energy efficiency from the first charge‐discharge cycle, followed by O_2_, Ar and H_2_, whereas C_2_H_2_ showed the lowest performance. This compares very well with the different capacities and the stabilities observed in the following section.

Thus, the crucial impact of gases on performance already occurs in the first charge‐discharge cycle. It must be noted that CO_2_ and C_2_H_2_ significantly alter the formation step and therefore the resulting SEI and its performance; CO_2_ has a positive impact and C_2_H_2_ a negative one.

### C‐rate test

**Figure 5:**
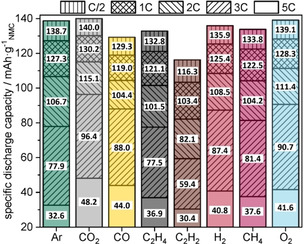
Specific mean discharge capacity observed during C‐rate testing for the cells with electrolyte saturated with various gases.

C‐rate capability tests were performed to further quantify the impact of gases on the total kinetic and transport losses during the discharge process. Figure 2 and the more detailed Figure 5 both show significant differences in discharge capacities during a C‐rate test for the various gases. The capacity values for the individual gases are given in Figure 5 and are discussed quantitatively in the following (for the normalized C‐rates and for battery cycling, see also Figure S2 and S3). Compared to the Ar reference, an improvement in C‐rate capability at low discharge currents was found for CO_2_ and O_2_. Furthermore, for CO_2_ an increase of 8 % at 2C, 24 % at 3C and even 48 % at 5C was achieved, compared to Ar. Moreover, O_2_ showed a significant increase in C‐rate capability: At 2C it had 4 %, at 3C 16 %, and at 5C 28 % more capacity than Ar. Nearly the same discharge capacities at 0.5/1/2C as those for Ar were found for CO, C_2_H_4_, H_2_ and CH_4_. Again, C_2_H_2_ showed the worst performance. In contrast to the lower C‐rates, where CO_2_ and O_2_ are the best, we can also see at 3C and 5C that CO (13 or 35 %) and H_2_ (12 or 25 %) have improved C‐rate capabilities. Notably, the gases, even if they have only a modest solubility (Ar<0.1 %; CO_2_≈1.1 %; C_2_H_4_≈1.1 %; H_2_<0.1 %; CH_4_≈0.2 %),[^52^, ^53^, ^54^, ^55^] exhibit substantial influence on the electrochemical properties as well as overall battery performance. Even Ar and H_2_ with their poor solubility show differences at 3C to 5C. The effect of the gases seems to be limited to kinetic or transport loss effects visible only at higher currents.

**Figure 6:**
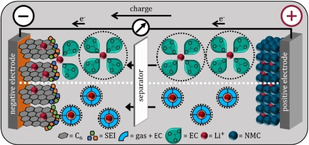
Schematic representation of the possible effect of gas saturation; different solvate shell radii due to the gas additives. Above: electrolyte without gases; below: electrolyte saturated with gases; circle: diameter of the inner and outer solvated shells.

Lithium ions do not migrate freely, but are surrounded by a “solvate shell”, and it is known that the type of solvate shell has a great influence on the mobility and the processes of the lithium ions.[^3^, ^56^, ^57^, ^58^] In the solvate shell, a network of electrolyte molecules is arranged around the lithium ion (Figure 6). Various dissolved gases in the electrolyte can have an influence on the formation of this solvate shell and thus possibly influence the mobility of lithium ions. Many factors can influence this, for example solubility concentrations, dipole moments, partial charges as well as coordination by free electron pairs, but a clear assignment is not possible at this point. For instance, diffusivity can be enhanced or hindered due to volume changes in the solvate shell in certain lithium ion coordination modes.^[59]^

In this respect, transport processes may be faster. Additionally, the removal of solvate shells is affected by the charge transfer before intercalation into the active material. To evaluate these theories, electrochemical impedance spectroscopy was used.

### Electrochemical impedance spectroscopy

Voltage losses can be classified into charge transport losses, charge transfer losses due to electrochemical reactions and losses due to reactant diffusion.

**Figure 7:**
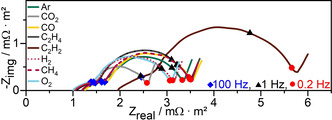
EIS measurements of full cells with different gas‐saturated electrolytes in the 4th cycle at SOC 50. Selected frequencies are marked.

By means of electrochemical impedance spectroscopy, the different time constants of these processes can be exploited to separate them and their respective contributions to the total loss and degradation of lithium‐ion batteries. Figure 7 shows the Nyquist plot of the measured gas‐electrolyte mixtures of the lithium‐ion batteries in the 4th cycle, directly after formation.

The recorded spectra can be divided into four areas: (1) the resistance at high frequencies (>1 kHz) contains the charge transport resistance from current collector, wiring, electrical and ionic transport through electrode and electrolyte, (2) a small semicircle in the high frequency range (1 kHz to 100 Hz) is often attributed to processes in the SEI or its interface, (3) a large semicircle in the mid frequency range (100 Hz to 0.2 Hz) that can be attributed to negative electrode/electrolyte and positive electrode/electrolyte interface double layers and to charge transfer processes, and (4) a monotonically increasing straight line at frequencies below 0.2 Hz, that is typically associated with lithium solid diffusion within the active material and other slow diffusion processes.[^60^, ^61^, ^62^, ^63^]

The total impedance magnitude |*Z*|, up to the diffusion‐dominated frequency<0.2 Hz, includes the cumulative contribution of the charge transport resistance, SEI, and charge transfer processes (for details, see Table S1). |*Z*| follows a similar trend to the charge–discharge tests: CO_2_ and O_2_ (2.54 and 3.05 mΩ m^−2^) show (after formation) the lowest impedances overall, as well as the best C‐rate capabilities and capacities.

The results for Ar and H_2_ (3.10 to 3.27 mΩ m^−2^), C_2_H_4_, CO, CH_4_ (3.44 to 3.46 mΩ m^−2^) and C_2_H_2_ (5.64 mΩ m^−2^) again follow the trend of the charge–discharge tests, with C_2_H_2_ showing by far the highest capacity losses. The gas effects on the impedance become clearer when separately comparing the regions. CO_2_, and O_2_ have the lowest resistances (ca. 1.00 mΩ m^−2^), followed by H_2_ and C_2_H_4_ (ca. 1.06 mΩ m^−2^), as well as CH_4_, Ar and CO (ca. 1.21 mΩ m^−2^) and C_2_H_2_ (1.96 mΩ m^−2^). The positive effects of the reactive gases compared to Ar support the hypothesis that lithium‐ion migration is improved by the contained gases (as described above).

The influences of the gas–electrolyte mixtures on the impedances of the SEI process are similar for all gases (approximately 0.48 to 0.67 mΩ m^−2^, except C_2_H_2_, which is 5.5 times larger). Much more dominant are the losses due to charge transfer at the electrodes, which are about 2 to 3 times larger than the SEI related impedances. The trend for the gas‐electrolyte mixtures is the same as for the previously discussed charge‐discharge capacities. This is clear evidence that the gases may influence the reaction overpotential, for example, through stripping of the solvate shell, or the availability and mobility of lithium ions at the electrode/SEI interface.^[64]^

**Figure 8:**
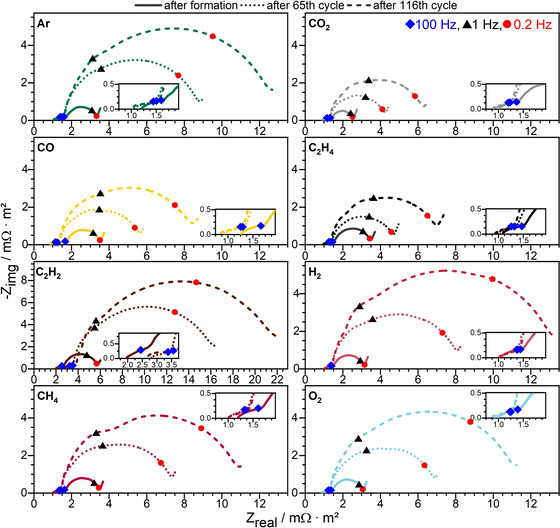
Evolution of EIS measurements on full cells of the different gas‐saturated electrolytes during aging: after formation, 65th and 116th cycles. For better visualization, the axes of the graph C_2_H_2_ have been scaled differently from the others.

Since large differences in impedance behavior were already observed after formation, we subsequently carried out analogous investigations on the aging behavior (Figure 8).

The impedance spectra at different aging times allow one to identify changing contributions – especially to high frequency resistance, SEI and charge transfer processes. Impedance after the 65th and 116th cycles were compared with those after formation. In general, the high frequency resistance slightly decreased or remained constant with increasing cycle number (except for C_2_H_2_, where the impedance increased by about one third after 65 cycles and then remained constant). During aging the impedances of the SEI processes decreased by about 10 to 15 % for all gases, which may be due to the improvement of SEI properties (e. g., morphological changes leading to a more compact and ionically conductive layer).[^27^, ^32^, ^33^, ^34^, ^35^, ^36^, ^37^] In contrast, large differences in the charge transfer processes due to aging were observed: an increase in the low frequency semicircle and the characteristic time constant. After 65 cycles, the |*Z*| at the lowest frequency of the charge transfer regime for Ar increased by 4.8 times, and after 116 cycles by more than 7 times, compared to the 4th cycle. In contrast, the same values for CO_2_ increased by only 2.5 times and 4.4 times after the 65th and 116th cycles, respectively. This clearly shows that CO_2_ saturated electrolyte has a much better performance and a lower aging behavior, both in EIS and in C‐rate capability (as well as capacities). CO and C_2_H_4_ showed a similar aging behavior in the charge transfer processes (about 1.5 times each after 65 and 116 cycles, compared to their 4th cycle), with CO showing a slightly stronger aging trend. In addition, CH_4_ and O_2_ show similar aging behavior to each other and the charge transfer processes after formation exhibit an increase of about 2 times after 65 and 116 cycles, compared to their 4th cycle. Whereas H_2_ showed one of the lowest values for the charge transfer processes after the 4th cycle, it showed the second most severe aging, followed by C_2_H_2_, which showed the worst performance and the most severe aging for all processes and investigations.

The results of the impedance spectroscopy confirm the positive effect of the CO_2_ saturated electrolyte on the performance of lithium‐ion batteries, especially related to kinetic performance and aging.

## Conclusion

In this work, we have shown that presaturation of electrolytes with different gases leads to strong and characteristic changes in the performance and cycling stability of lithium‐ion batteries. Ar, CO_2_, CO, C_2_H_4_, C_2_H_2_, H_2_, CH_4_, and O_2_ showed characteristic impacts on all measurements, including on charge and discharge characteristics, C‐rate capabilities, and impedance behavior.

The different gases led to changes in the electrochemical processes that occur during formation, observed through differences in the electrical charge consumed during the first cycle and through differences in potential behavior. The best results were found for CO_2_, not only in SEI formation, but also in the charge–discharge and C‐rate tests. Compared to the reference cell with Ar and all other gas‐saturated electrolytes, a higher C‐rate capability and a decrease in irreversible loss were observed for CO_2_. This clearly shows that CO_2_‐saturated electrolytes lead to an increase in lithium‐ion battery performance and that the influence of gases must not be neglected. The CO_2_‐saturated electrolyte also showed the best properties in terms of aging behavior, with the lowest capacity loss of all gases and an 9 % higher capacity retention compared to the Ar reference. A comparable trend was observed in electrochemical impedance spectroscopy, where CO_2_ showed significantly lower impedance in both total impedance in charge transport and charge transfer processes. This trend became even more dominant during aging. Whereas only minor differences were observed for the charge transport resistance at high frequency and the SEI processes, the changes in the charge transfer were significantly affected. Besides the CO_2_ behavior, C_2_H_4_ and CO also showed low impedances during aging, whereas the other gases (including Ar) exhibited significantly higher increases. This proves that the dissolved gases cause an additional, significant influence on the charge transfer processes in lithium‐ion batteries.

The results lead to the conclusion that gases, especially CO_2_ and C_2_H_2_, have a significant influence on battery behavior. Further systematic studies concerning gas influences on batteries need to be conducted to confirm the proposed underlying causes, especially utilizing surface sensitive methods to identify differences in electrode surface film morphology. We believe that a better understanding of gas–electrolyte influences can lead to further improvement approaches in the development of high‐performance lithium‐ion batteries, battery safety, or fast charging. Specifically, pre‐gassing an electrolyte with CO_2_ might be used to improve battery performance and lifetime.

## Experimental Section

### Materials and equipment

For all experiments, a three‐electrode PAT‐Cell setup (EL‐Cell GmbH) was used, with a lithium ring as reference electrode. Electrodes were purchased from CustomCells Itzehoe GmbH. The negative electrode was composed of 96 wt % active material (graphite, SMG104) and 4 wt % of additives including binder (sodium carboxymethyl cellulose, Na‐CMC; styrene butadiene rubber, SBR) and conducting additive on copper foil. The active material had a specific capacity of 350 mAh g^−1^ and the composite had an area specific capacity of 2.2 mAh cm^−2^. The positive electrode was composed of 86 wt % of nickel manganese cobalt oxide (NMC622) and 14 wt % of additives including binder (poly vinylidene difluoride, PVdF) and conducting additive on aluminum foil. The active material had a specific capacity of 165 mAh g^−1^ and the composite had an area specific capacity of 2.0 mAh cm^−2^. All electrodes were punched out to receive a standard circle with a uniform diameter of 18 mm, their weight was measured (XS205, Mettler Toledo) and they were dried overnight at 120 °C under high vacuum before being transferred into an Ar glovebox (water and oxygen content under 0.1 ppm). All capacities were calculated based on the measured weight of assembled electrodes. The separator composition was as follows: double layered polypropylene (PP, Freudenberg FS 2226 E)/polyethylene (PE, Lydall Solupor 5P09B) with an approximate thickness of 220 μm and a porosity of 67 % (PP) and 86 % (PE; EL‐Cell GmbH; ECC1‐00‐0210V/X). Test cells were assembled from these materials and were filled with 100 μL of gas‐saturated electrolyte (EC/DMC 1 : 1 v/v, 1 m LiPF_6_, battery grade, Sigma‐Aldrich). All gases were purchased from Westfalen AG. The purities of the gases were: Ar (99.996 vol. %), CO_2_ (99.999 vol. %), CO (99.999 vol. %), C_2_H_4_ (99.95 vol. %), C_2_H_2_ (99 vol. %), H_2_ (99.999 vol. %), CH_4_ (99.95 vol. %) and O_2_ (99.999 vol. %). The gas‐saturated electrolytes were prepared as follows: In an Ar glovebox, 7 mL electrolyte were inserted into a 22.4 mL headspace vial for each sample and then the vial sealed gastight. The subsequent gas saturations were carried out outside the glovebox under water and air free conditions by mounting a syringe in‐ and outlet onto the vial and bubbling the gas directly into the electrolyte for 15 min at 23 °C and releasing the excess gas via the headspace (Figure S4). To avoid atmospheric impurities, gas supply lines were thoroughly flushed with the respective gas before use and a slight overpressure was applied. Please note, it is important to ensure a slight overpressure in the vial so that no atmospheric components can enter. Before the electrolyte was saturated with the individual gases, the electrolyte had been stored by the manufacturer under Ar. All cells were cycled with a Maccor potentiostat (Maccor Inc., Series 4000) and EIS‐measurements were conducted with a Gamry reference 3000 with auxiliary electrometer (Gamry Instruments) in a temperature chamber (Espec Europe GmbH, SU 642) at 25 °C.

### Electrochemical conditioning and characterization

The formation‐and‐cycling protocol was identical for all experiments and is listed in Table 1. All potential values are given vs. Li/Li^+^ as reference electrode. The voltage range was kept between 2.9 V and 4.2 V.

**Table 1:** Cycling procedure.

	Parameter	Value	
	Temperature	25 °C	
	Formation	C/10	
	C‐rate test	[0.5/1/2/3/5]C	
	Cycle	1C	
	Charge	CC/CV	
	Discharge	CC	
	*U*_cutoff_	4.2 V/2.9 V

After cell assembly and a 10 h rest time, the cells were formatted two times with C/10 in a CC (constant current) step for charge and a CC/CV (constant current/constant voltage) step for discharge. Constant voltage (dis‐)charging was stopped when current was equal to or less than C/20; the final value was used to determine the capacity and to set the charge–discharge current for the respective C‐rate. Following the formation step, a discharge C‐rate test was conducted for in‐depth characterization, whereby the cells were discharged twice with [0.5/1/2/3/5]C in a CC step and charged with 1C in a CC/CV step. Then the cells were aged by carrying out two sets of cycling procedures, each with 50 cycles at 1C, separated by an impedance measurement (EIS). Charging was performed with CC/CV and discharging with CC, followed by 10 min rest time. EIS was measured at a state of charge (SOC) of 50 % after formation, just after the 65th cycle (includes C‐rate test) and at the end after the 116th cycle. The EIS‐protocol was identical for all experiments and is listed in Table 2. To guarantee and quantify reproducibility, four measurements were conducted for C_2_H_2_, with three measurements for CO_2_, CO, C_2_H_4_, H_2_, CH_4_, and O_2_, and two measurements for Ar.

**Table 2:** Parameters of EIS‐experiments.

	Parameter	Value	
	Temperature	25 °C	
	Mode	galvanostatic	
	AC current	5×10^−4^ A_rms_	
	Frequency	2×10^−2^ to 10^5^ Hz	
	Points/decade	10

## Conflict of interest

The authors declare no conflict of interest.

## Supporting information

As a service to our authors and readers, this journal provides supporting information supplied by the authors. Such materials are peer reviewed and may be re‐organized for online delivery, but are not copy‐edited or typeset. Technical support issues arising from supporting information (other than missing files) should be addressed to the authors.

Supporting Information
