# Supplementary material for: The Effects of Gas Saturation of Electrolytes on the Performance and Durability of Lithium‐Ion Batteries
Source: ChemSusChem. 2021 Jun 16;14(14):2943–51. doi: 10.1002/cssc.202100845 (PMC8361957; doi:10.1002/cssc.202100845)
Supplement: Supplementary file 1 — Supporting Information [file CSSC-14-2943-s001.pdf]

ChemSusChem

Supporting Information

The Effects of Gas Saturation of Electrolytes on the Performance and Durability of Lithium-Ion Batteries

Lars Bläubaum, Philipp Röse, Leon Schmidt, and Ulrike Krewer*© 2021 The Authors. ChemSusChem published by Wiley-VCH GmbH. This is an open access article under the terms of the Creative Commons Attribution License, which permits use, distribution and reproduction in any medium, provided the original work is properly cited.

Table of Content

Figure S1: Positive electrode potential vs. charge/discharge capacity p. 2
Figure S2: Normalized discharge capacities observed during the C-rate test after formation p. 2
Figure S3: Normalized discharge capacity during cycling p. 3
Figure S4: Schematic structure for carrying out the gas saturation p. 3
Table S1: EIS-Data p. 4

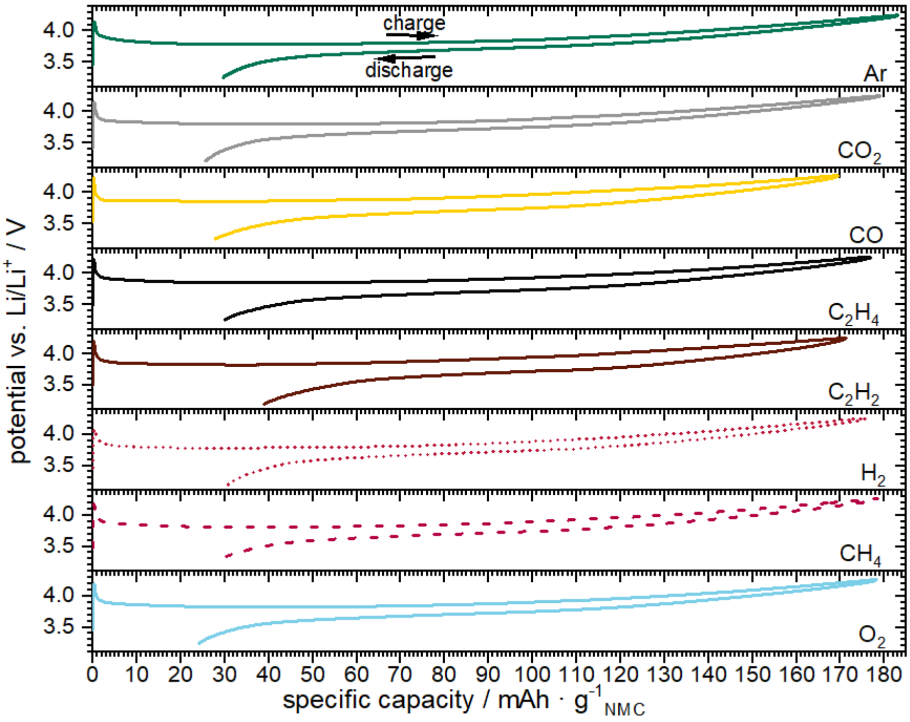

Figure S1. Positive electrode potential vs. charge/discharge capacity, first C/10 formation step for cell electrolytes saturated with different gases.

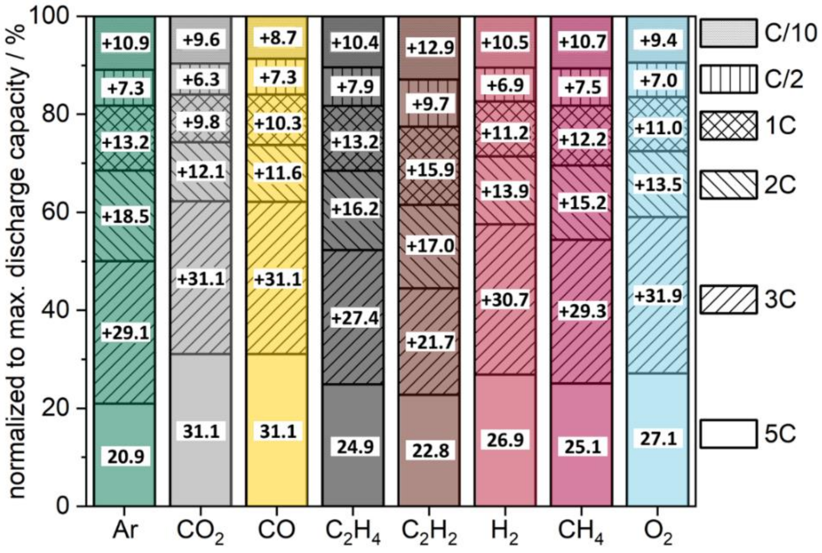

Figure S2. Discharge capacities observed during the C-rate test after formation; normalized to maximum discharge capacity at C/2 results are displayed for cells with electrolyte saturated with various gases.

To identify different kinetic behavior depending on the current load and the available capacity, the capacities of the different C-rates were normalized by the capacity for C/10 (Figure S2). The following gas pairs (1) CO_2 and CO, (2) C_2H_4 and CH_4, (3) H_2 and O_2 showed similar behavior. The strong dependence of kinetic behavior on gas saturation reinforces our assumption that gases influence electrochemical reaction taking place during formation. These gases appeared to provide the lowest losses and thus the best C-rate opportunities. Overall, C_2H_2 showed a reduction in all C-rates. For detailed discussion of the results, we refer to the manuscript.

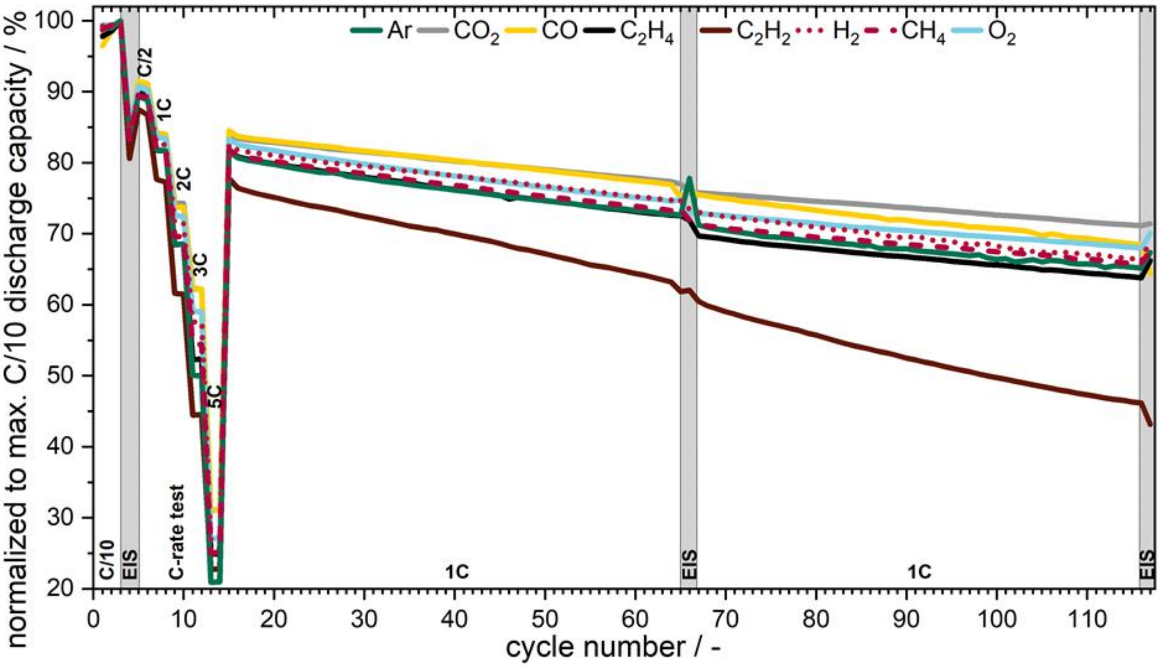

Figure S3. Normalized discharge capacity; normalized to maximum discharge capacity of the 3rd C/10-cycle; results are displayed for cells with electrolyte saturated with various gases.

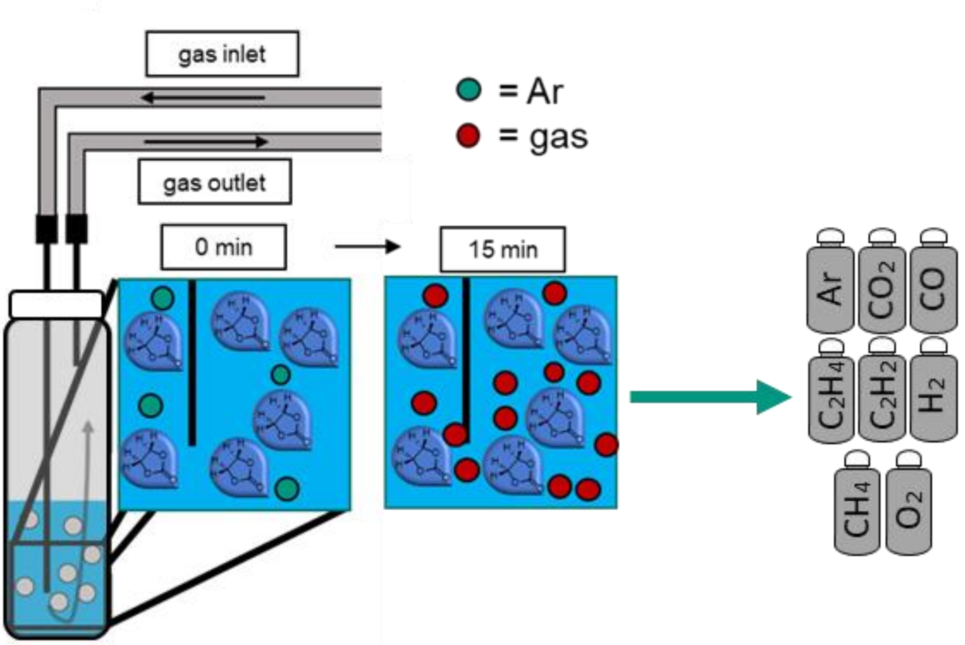

Figure S4. Schematic structure for carrying out the gas saturation.

Table S1. Calculated EIS-Data after formation (4th, cycle, 1st), after 65th cycle (2nd) and after 116th cycle (3rd).

Gas	Step	R_{Bulk} / mΩ m²	R_{SEI} / mΩ m²	R_{CT} / mΩ m²
Ar	Formation	1.20	0.67	1.40
	65th cycle	1.04	0.62	6.68
	116th cycle	0.99	0.69	10.31
CO_2	Formation	1.01	0.48	1.06
	65th cycle	0.93	0.45	2.68
	116th cycle	0.90	0.44	4.62
CO	Formation	1.25	0.62	1.58
	65th cycle	0.92	0.53	3.91
	116th cycle	0.86	0.49	6.52
C_2H_4	Formation	1.07	0.56	1.81
	65th cycle	0.98	0.51	3.07
	116th cycle	0.94	0.46	5.26
C_2H_2	Formation	1.96	2.62	1.06
	65th cycle	2.83	0.77	12.02
	116th cycle	2.72	1.18	16.84
H_2	Formation	1.04	0.57	1.49
	65th cycle	1.03	0.40	6.49
	116th cycle	1.05	0.42	11.06
CH_4	Formation	1.19	0.59	1.66
	65th cycle	1.01	0.42	5.56
	116th cycle	1.04	0.39	8.98
O_2	Formation	0.99	0.57	1.49
	65th cycle	0.91	0.49	5.38
	116th cycle	0.84	0.66	9.45
